# Supplementary material for: Inducible Nitric Oxide Synthase (iNOS) Mediates Vascular Endothelial Cell Apoptosis in Grass Carp Reovirus (GCRV)-Induced Hemorrhage
Source: Int J Mol Sci. 2019 Dec 16;20(24):6335. doi: 10.3390/ijms20246335 (PMC6941106; doi:10.3390/ijms20246335)
Supplement: Supplementary file 1 [file ijms-20-06335-s001.pdf]

Supplementary Materials

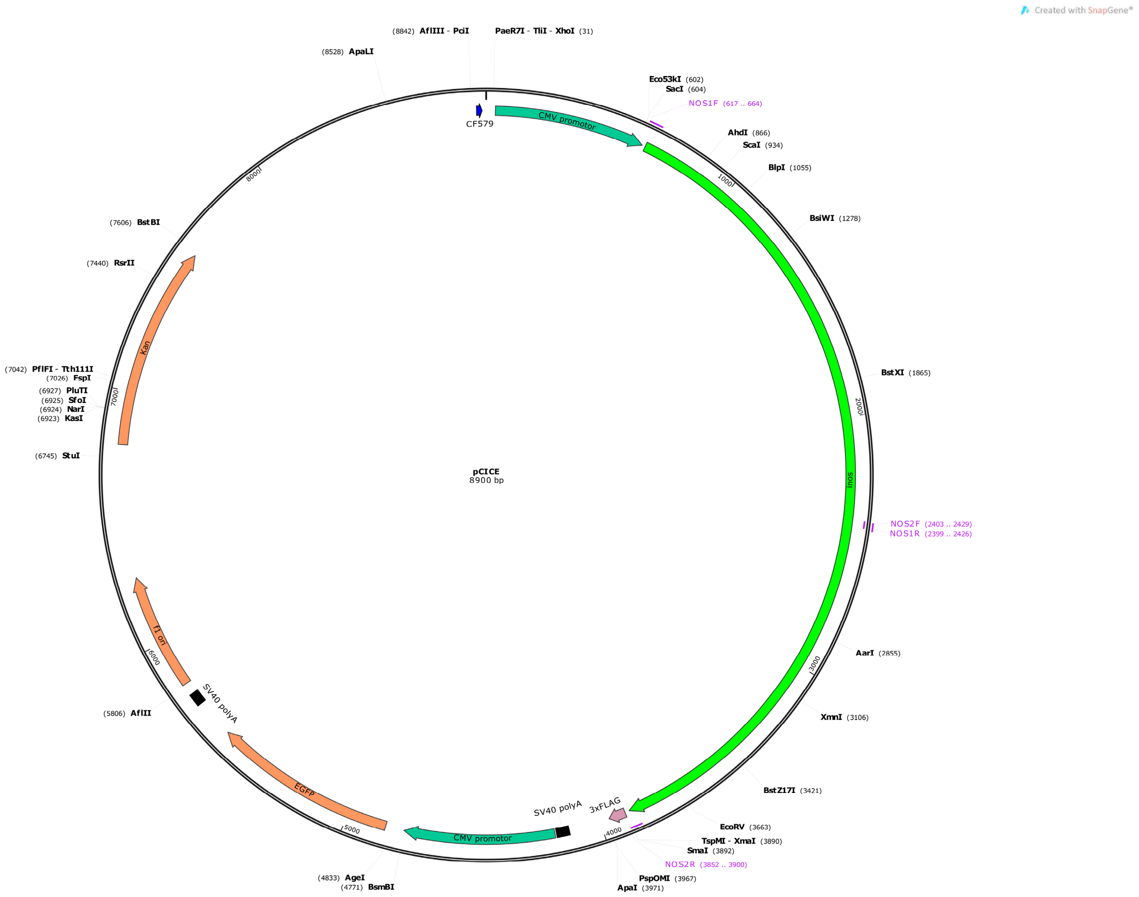

Figure S1. The structure of plasmid CMV-*iNOS*-3×Flag-CMV-eGFP.

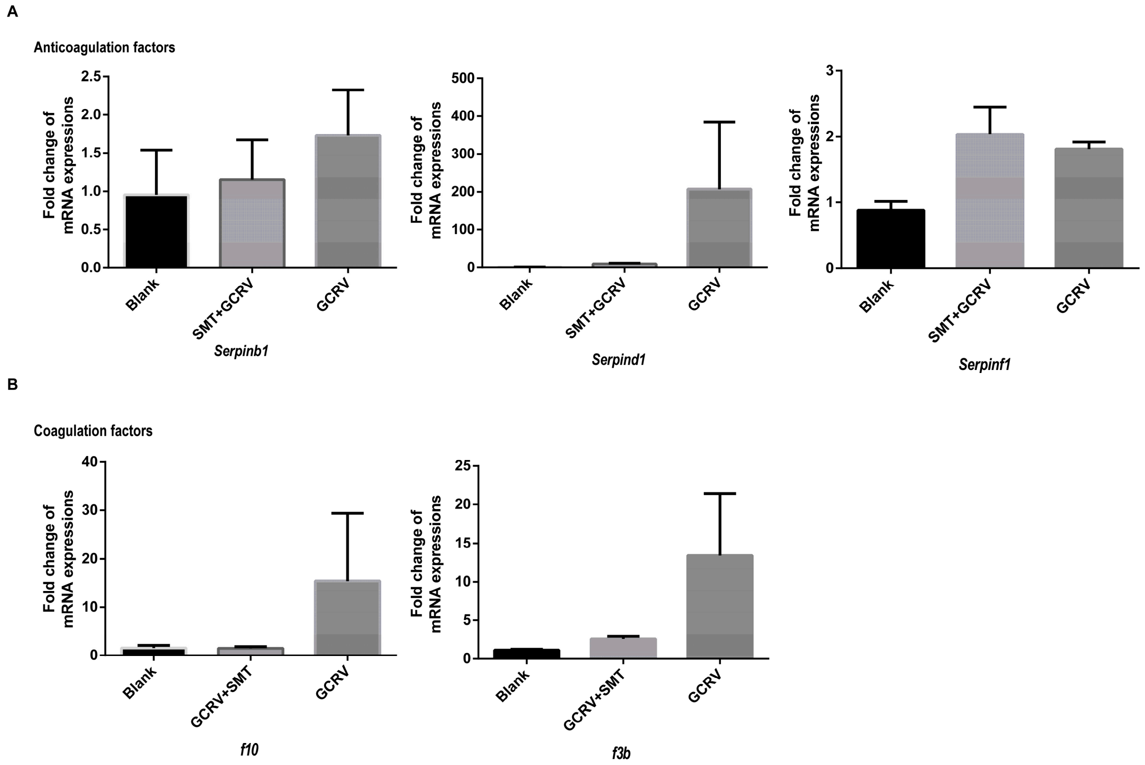

Figure S2. Factors with no significant changes in coagulation and anticoagulation systems. (**A**) Anticoagulation factors: *serpinb1*, *serpind1*, and *serpinf1* (*n*=3). (**B**) Coagulation factors: *f10* and *f3b* (*n*=3).

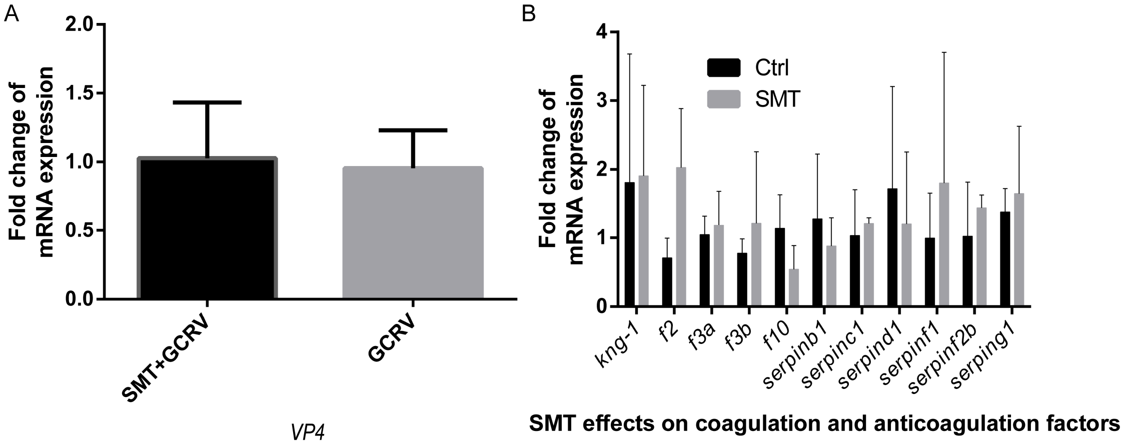

Figure S3. SMT has no direct effect on GCRV replication, coagulation and anticoagulation factors. Samples were collected on the fifth day after GCRV infection and the concentration of SMT is 100 mg/kg. (**A**) *VP4* is one of the GCRV structural proteins, which was used to detect GCRV replication in *vivo* (*n*=3). (**B**) Coagulation and anticoagulation factors were detected in *vivo* (*n*=3).
